# Seroprevalence of HTLV‐1 and HTLV‐2 amongst mothers and children in Malawi within the context of a systematic review and meta‐analysis of HTLV seroprevalence in Africa

**DOI:** 10.1111/tmi.12659

**Published:** 2016-02-03

**Authors:** James M. Fox, Nora Mutalima, Elizabeth Molyneux, Lucy M. Carpenter, Graham P. Taylor, Martin Bland, Robert Newton, Fabiola Martin

**Affiliations:** ^1^ Centre for Immunology and InfectionDepartment of Biology and Hull York Medical SchoolUniversity of YorkYorkUK; ^2^ Health SciencesUniversity of YorkYorkUK; ^3^ Department of Orthopaedic SurgeryMonash HealthMelbourneAustralia; ^4^ Paediatric DepartmentCollege of MedicineQueen Elizabeth Central HospitalBlantyreMalawi; ^5^ Nuffield CollegeUniversity of OxfordOxfordUK; ^6^ National Centre for Human Retrovirology/HTLV clinicImperial College Healthcare NHS TrustSt Mary's HospitalLondonUK; ^7^ MRC/UVRI Uganda Research Unit on AIDSEntebbeUganda

**Keywords:** Africa, Human T‐lymphotropic virus, Malawi, prevalence, seroprevalence, mother‐to‐child transmission, MTCT, HTLV‐1, HTLV‐2, systematic review, meta‐analysis, healthy women, Afrique, HTLV, Malawi, prévalence, séroprévalence, transmission mère‐enfant, TME, virus T‐lymphotropique humain, HTLV‐2, revue systématique, méta‐analyse, femmes en bonne santé

## Abstract

**Objectives:**

Human T‐lymphotropic virus (HTLV)‐1 causes T‐cell leukaemia and myelopathy. Together with HTLV‐2, it is endemic in some African nations. Seroprevalence data from Malawi are scarce, with no reports on associated disease incidence. HTLV seroprevalence and type were tested in 418 healthy mothers from Malawi. In addition, we tested the sera of 534 children to investigate mother‐to‐child transmission. To provide context, we conducted a systematic review and meta‐analysis of HTLV seroprevalence in African women and children.

**Methods:**

Stored samples from a previous childhood cancer and BBV study were analysed. ELISA was used for HTLV screening followed by immunoblot for confirmation and typing. Standard methods were used for the systematic review.

**Results:**

HTLV seroprevalence was 2.6% (11/418) in mothers and 2.2% (12/534) in children. Three mothers carried HTLV‐1 alone, seven had HTLV‐2 and one was dually infected. Three children carried HTLV‐1 alone, seven had HTLV‐2 and two were dually infected. Only two corresponding mothers of the 12 HTLV‐positive children were HTLV positive. The systematic review included 66 studies of women and 13 of children conducted in 25 African countries. Seroprevalence of HTLV‐1 varied from 0 to 17% and of HTLV‐2 from 0 to 4%.

**Conclusions:**

In contrast to findings from other studies in Africa, the seroprevalence of HTLV‐2 was higher than that of HTLV‐1 in Malawi and one of the highest for the African region. The lack of mother–child concordance suggests alternative sources of infection among children. Our data and analyses contribute to HTLV prevalence mapping in Africa.

## Abstract (fr)

**Objectifs:**

Le virus T‐lymphotropique humain (HTLV) ‐1 provoque la leucémie des lymphocytes T et la myélopathie. Ensemble avec HTLV‐2, il est endémique dans certains pays africains. Les données de séroprévalence du Malawi sont rares, avec aucun report sur l'incidence de la maladie associée. La séroprévalence et le type de HTLV ont été testés chez 418 mères en bonne santé du Malawi. En outre, nous avons testé les sérums de 534 enfants pour investiguer sur la transmission mère‐enfant. Afin de fournir un contexte, nous avons effectué une revue systématique et une méta‐analyse de la séroprévalence du HTLV chez les femmes et les enfants africains.

**Méthodes:**

Des échantillons stockés provenant de cancer de l'enfance et de l’étude BBV ont été analysés. Le test ELISA a été utilisé pour le dépistage du HTLV suivi par l'immunoblot pour la confirmation et le typage. Des méthodes standard ont été utilisées pour l'analyse systématique.

**Résultats:**

La séroprévalence du HTLV était de 2,6% (11/418) chez les mères et de 2,2% (12/534) chez les enfants. Trois mères portaient HTLV‐1 seul, sept avaient HTLV‐2 et une était doublement infectée. Trois enfants portaient HTLV‐1 seul, sept avaient HTLV‐2 et deux étaient doublement infectés. Seules deux mères correspondantes chez les 12 HTLV enfants séropositifs étaient positives pour HTLV. L'analyse systématique a inclus 66 études sur des femmes et 13 sur des enfants, menées dans 25 pays africains. La séroprévalence du HTLV‐1 variait de 0 à 17% et celle de HTLV‐2 de 0 à 4%.

**Conclusions:**

Contrairement aux résultats d'autres études en Afrique, la séroprévalence du HTLV‐2 était plus élevée que celle du HTLV‐1 au Malawi et une des plus élevées de la région africaine. Le manque de concordance mère‐enfant suggère d'autres sources de l'infection chez les enfants. Nos données et les analyses contribuent à la cartographie de la prévalence du HTLV en Afrique.

## Abstract (es)

**Objetivos:**

El virus linfotrópico humano de células T (HTLV)‐1 causa leucemia de las células T y mielopatía. Junto con HTLV‐2 es endémico en algunas naciones africanas. Los datos de seroprevalencia para Malawi son escasos, sin informes sobre la incidencia de la patología asociada. Se evaluó la seroprevalencia y el tipo de HTLV en 418 madres sanas de Malawi. Adicionalmente evaluamos el suero de 534 niños para investigar la transmisión madre‐hijo. Para ponerlo en contexto, realizamos una revisión sistemática y metaanálisis de seroprevalencia de HTLV en mujeres y niños africanos.

**Métodos:**

Se analizaron muestras de un estudio previo de cáncer infantil y estudio BBV. Se utilizó la prueba de ELISA para el cribado de HTLV seguido por un inmunoblot para realizar la confirmación y el tipado. Se utilizaron metodologías estándar para la revisión sistemática.

**Resultados:**

La seroprevalencia de HTLV era del 2.6% (11/418) en madres y 2.2% (12/534) en niños. Tres madres tenían el HTLV‐1 solamente, siete tenían HTLV‐2 y una estaba infectada con ambos. Tres niños estaban infectados con HTLV‐1 solamente, siete tenían HTLV‐2 y dos estaban infectados con ambos. Solo dos madres de los 12 niños HTLV positivos eran HTLV positivas. La revisión sistemática incluyó 66 estudios de mujeres y 13 de niños realizados en 25 países africanos. La seroprevalencia de HTLV‐1 variaba entre 0‐17% y de HTLV‐2 entre 0‐4%.

**Conclusiones:**

En contraste con los hallazgos de otros estudios en África, en Malawi la seroprevalencia de HTLV‐2 era mayor que la de HTLV‐1, y una de las más altas para regiones africanas. La falta de concordancia en la transmisión madre‐hijo sugiere la existencia de fuentes alternativas de infección entre niños. Nuestros datos y análisis contribuyen a mapear la prevalencia de HTLV en África.

## Introduction

HTLV‐1 is the causative agent of aggressive adult T‐cell leukaemia/lymphoma (ATLL) and the progressive, chronic, disabling HTLV‐1‐associated myelopathy/tropical spastic paraparesis (HAM/TSP) as well as other inflammatory conditions such as infective dermatitis and uveitis 1. Surveillance data for prevalence of these two conditions are scarce and virtually unavailable for large areas such as China, India and many African countries. Even fewer data are available on prevalence and pathogenicity of HTLV‐2, which is known to be endemic in African Pygmies 2 and Native Americans 3. In the United States, HTLV‐2 is more common than HTLV‐1 and is associated with female sex, older age, Asian, Hispanic and African ethnicity, low level of education, a history of injecting drug use in the 1960s/1970s (a result of birth cohort effects) and residence in western and south‐western USA, where HTLV‐2 clusters are found 4. There are case reports describing associations between HTLV‐2 and a small increased risk of bacterial infections, particularly of the chest and bladder 5, increased cancer risk 6 and rare reports of HAM/TSP 7.

HTLV‐1, an oncogenic human RNA retrovirus, was discovered 35 years ago 8, followed by the identification of HTLV‐2, HTLV‐3 and HTLV‐4 9, 10, 11. These human viruses have arisen through inter‐species transmission of simian T‐lymphotropic viruses (STLV) 12. HTLV‐1 is found in endemic clusters and an estimated 10–20 million individuals, worldwide, are infected 2, 13. Virus is transmitted from human‐to‐human through infected lymphocytes and may be acquired through mother‐to‐child transmission (MTCT): at birth and more commonly through breastfeeding, sexual intercourse, blood transfusions, organ transplantations and contaminated needle reuse.

The African continent has a population over 1 billion and represents the largest endemic area for HTLV infection but with many data gaps. HTLV seroprevalence has been reported only once in Malawi, at 2.5%, when 159 blood donor sera were screened by ELISA, without typing or confirmatory testing 14. HTLV associated diseases have not been reported in Malawi, which may be due to limited diagnosis, lack of surveillance and poor survival.

This study investigated the prevalence of anti‐HTLV‐1 and HTLV‐2 antibodies in stored sera from a previous childhood cancer study. We had access to the sera of 534 children diagnosed with cancer in Blantyre, Malawi and 418 paired healthy mothers. ELISA positive tests were confirmed and typed by immunoblotting. In addition, we applied context to our results by conducting a systematic review and meta‐analysis of published HTLV seroprevalence data of African women and children.

## Methodology

### Study population

Anonymised stored serum samples from Malawian children (*n* = 534) and, where available, from their biological mothers (*n* = 418), were tested. Samples had been originally collected as part of a childhood malignancy and blood‐borne virus (BBV) study at the Queen Elizabeth Central Hospital in Blantyre, Malawi between 2006 and 2010 15. Their mothers were the children's healthy controls as part of this original study. Ethical approval for the study was obtained from the Oxford Tropical Research Ethics Committee and the Malawian College of Medicine Research and Ethics Committee. Written informed consent was obtained from mothers and the guardians of the children. Details of the original study are published elsewhere 15. Demographic data were not available for all children (minimum *n* = 174, maximum *n* = 276) or mothers (minimum *n* = 140, maximum *n* = 209), and data were not available for all data points. Results are, therefore, presented as % of data available.

### Screening, confirmation and typing of HTLV

Replicating clinical diagnostic algorithms, the study protocol required that all ELISA reactive samples underwent immunoblot (IB) confirmatory testing. Only indeterminate IBs were tested further with polymerase chain reaction (PCR), where whole blood was available. All sera were screened in duplicate by ELISA (MP Diagnostics HTLV‐1/2 ELISA v4, antibodies against gp46‐I, gp46‐II, GD21) following the manufacturers' instructions (MP Biomedicals, Cambridge, Cambridgeshire, UK). ELISA reactive or borderline reactive sera were tested and typed as HTLV‐1, HTLV‐2, HTLV‐1 and HTLV‐2, negative and indeterminate using an FDA approved, confirmatory, qualitative enzyme immunoblot assay (IB), (MP Diagnostics HTLV blot v2.4), which were interpreted using the HTLV European Research Network guidelines 16, 17. Both assays included HTLV‐positive serum controls provided by the manufacturer. Where frozen whole blood was available, samples typed as indeterminate were further tested for the presence of HTLV DNA (*n* = 18). DNA was isolated from frozen whole blood of serologically indeterminate samples and its integrity was confirmed by optimised PCR amplification of glyceraldehyde 3‐phosphate dehydrogenase (GAPDH) using commercially available primers. Samples were tested for the presence of HTLV‐1 DNA by PCR as previously described 18.

### Systematic review

PRISMA guidelines for systematic reviews and meta‐analyses were adhered to during protocol design and analyses. The NCBI PubMed database was searched using the search terms ‘HTLV’, ‘human T‐lymphotropic virus’, ‘mother’, ‘child’, ‘transmission’ plus each respective African nation to 31 December 2014.

**Figure 1:**
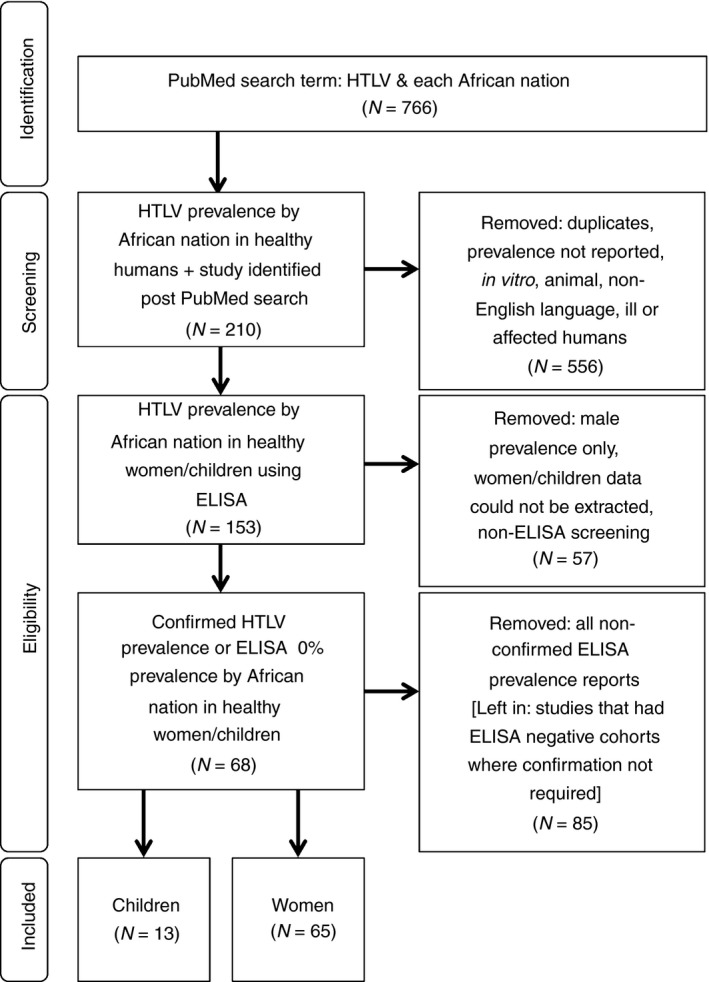
Flow chart of all manuscripts screened for prevalence of HTLV in healthy women and children living in Africa. Ten publications had data for both women and children.

The inclusion criteria were cross‐sectional seroprevalence studies; healthy women (to match our cohort, including blood donors, ante‐natal clinic attendees, healthy volunteers); sex workers; children; living in Africa; opt‐in, opt‐out or anonymous un‐linked (blood tests/other body fluid); approved by ethics; screened for HTLV; and confirmed with a confirmatory test (CT), and studies with zero seroprevalence by ELISA were included (Figure 1). The exclusion criteria were case reports; co‐infection studies in an already known infected population; ill patients (hospitalised, patients with underlying diagnosis); no CT; men; seroprevalence by gender unspecified; patient self‐reporting; and not in English language (Figure 1).

Applying the above criteria at stage 1 all potential papers' titles and abstracts were screened, and at stage 2 duplicates and papers not fitting all criteria were removed (Figure 1). Data were collected in Excel: location, year of publication, authors, study title, study characteristics, cohort gender, sample size, seroprevalence using ELISA, seroprevalence using confirmatory test and PCR confirmed. All citations on included papers were checked for any additional studies with one additional study identified not in the original PubMed searches (Figure 1). Data were screened and extracted by JF and checked by FM and MB. Any discordance was resolved through discussion.

### Meta‐analysis

The confidence intervals for individual studies were calculated by the exact binomial method and the combined estimate by random effects meta‐analysis using the logistic transformation. Where there were no observed HTLV cases, giving zero seroprevalence, 0.5 was added to frequencies for the meta‐analysis. There were much variability between studies; therefore, they all received similar weights in random effects meta‐analysis. Forest plots were created from the results of the meta‐analysis: squares represent the prevalence's reported for the individual studies, square areas being proportional to the weight the study received in the meta‐analysis. Horizontal lines show the 95% confidence intervals for the prevalence. The diamond shapes represent the combined estimates for each region, the deepest point being the estimated combined prevalence and the width of the diamond representing the 95% confidence interval. The confidence intervals were calculated on the log odds scale and transformed back to the proportion scale, and so they are not symmetrical. The data presented here were incorporated into the final analyses to give regional HTLV seroprevalence estimates.

## Results

### Demographics

Several data points were collected as part of the original publication 15, and we summarised it to have an overall picture of the cohorts' socio‐economical and health status; universal data were not available. 40% of children were female; mean age was 7.3 years (range 0.2–16). A total of 48% of children had Burkitt's, 12% neuroblastoma, none had ATLL. Only 12% of the mothers of the children had an illness during pregnancy, only 5.6% needed an intervention during delivery (caesarean section, stitches or assistance), 68% were delivered at home, only 4% were preterm deliveries. After birth, 98% were breastfed, 34% ≥2 years, 11% had blood transfusions, 51% had ever received an injection and 18% had a hospital admission before sampling; 81% had malaria in the past and 9% were HIV positive.

A total of 76% of mothers were married, 48% had >5 pregnancies, 45% had received injectable contraception, 36% reported to have had >1 partner, 9% reported having their first sexual intercourse during primary schooling and 34% during secondary school, 99% reported never having engaged in sex work, 94% never used condoms and 26% were HIV positive. A total of 81% of households had no electricity and 79% of children had to share plates with family members.

### Screening for HTLV prevalence in Malawian women and children

Twenty‐seven mothers' sera samples were repeatedly ELISA reactive and one was borderline reactive. Ten of 28 were HTLV‐positive by IB of which two were HTLV‐1 positive, seven HTLV‐2 and one was positive for both anti‐HTLV‐1 and anti‐HTLV‐2 antibodies (Table 1). HTLV‐1‐specific DNA was amplified by PCR from whole blood from 1/18 IB indeterminate participants; no HTLV‐1 or HTLV‐2‐specific DNA could be amplified from the remaining 17 indeterminate samples.

**Table 1:** HTLV seroprevalence in Malawi: screening of 418 mothers and 534 children and results of confirmatory testing of ELISA‐positive samples

	*N*	HTLV‐1 (%)	HTLV‐2 (%)	HTLV‐1 and HTLV‐2 (%)	Total (%)
Mothers	418	3 (0.7)	7 (1.7)	1 (0.2)	11 (2.6)
Children	534	3 (0.6)	7 (1.3)	2 (0.4)	12 (2.2)

Therefore, the final HTLV prevalence in 418 healthy women was 2.6%: three mothers were typed HTLV‐1 (0.7%) and seven HTLV‐2 (1.7%) with one (0.2%) mother being dually infected (Table 1).

Of 534 children, 82 sera were ELISA reactive and 12 by IB; three were typed HTLV‐1, seven HTLV‐2 and two HTLV‐1 and HTLV‐2 (Table 1). Only two children of HTLV‐positive mothers were also confirmed to be HTLV positive: the child of the HTLV‐1 and HTLV‐2 mother was HTLV‐2 positive and the child of a mother with HTLV‐1 was HTLV‐1 and HLTV‐2 positive. The mothers of the remaining 10 HTLV‐positive children were all HTLV seronegative. Whole blood was not available for any child.

In summary, the HTLV seroprevalence in children was 2.2%: 0.6% for HTLV‐1, 1.3% for HTLV‐2 and 0.4% for HTLV‐1/2 infection (Table 1).

Demographic data were not available for all the HTLV‐positive cases. However, where data were available, none of the HTLV‐positive children or mothers were HIV coinfected. Otherwise they resembled the rest of the HTLV‐negative cohort. Mothers were married, had never used condoms, had never engaged in sex work, had > 5 pregnancies and only one had received injectable contraception. Children's mean age was 8 years (range: 2.5–13.4 years), had been born vaginally, had not been born prematurely, had been breastfed for >2 years. Only one child had received injections in the past but none had had a blood transfusion and only three had a history of malaria.

### Systematic review and meta‐analysis of HTLV prevalence in Africa

**Figure 2:**
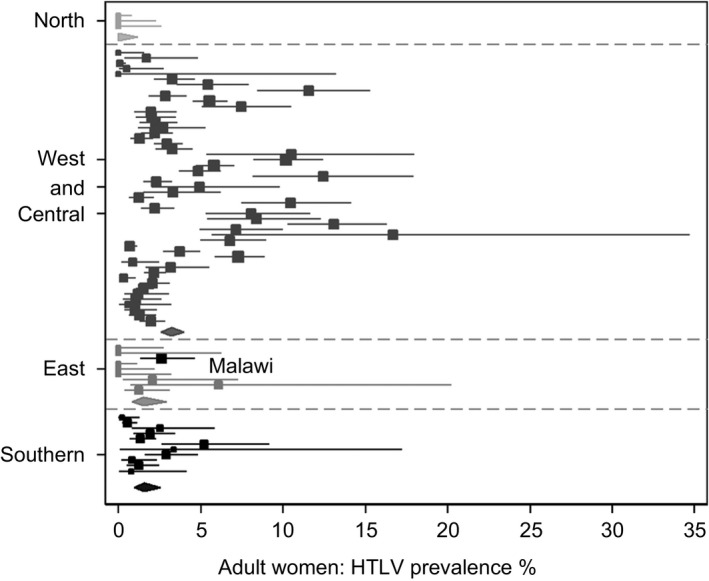
Forest plot showing prevalence of HTLV‐1 and HTLV‐2 among women, as reported in four regions of Africa, including data from Malawi. Squares represent each individual study prevalence with its area being proportional to the weight the study received in the meta‐analysis and horizontal lines showing the 95% CI's. Diamonds represent the combined estimates for each region with its width representing the 95% CI.

**Figure 3:**
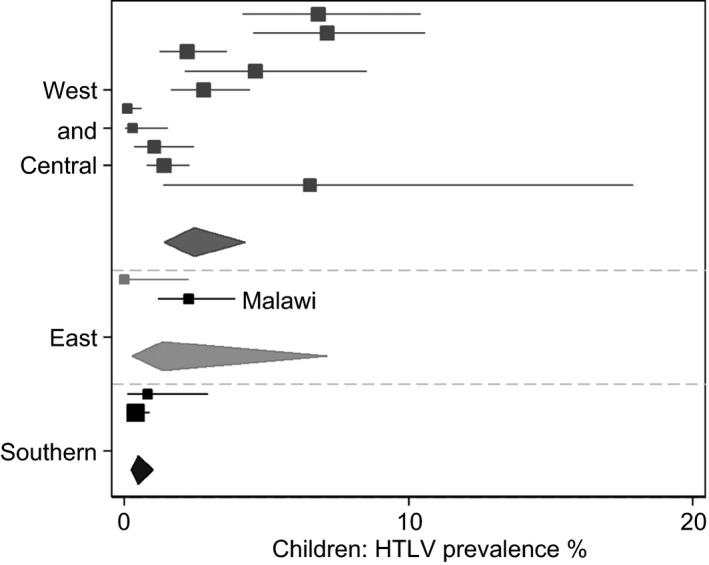
Forest plot showing prevalence of HTLV‐1 and HTLV‐2 among children, as reported in three regions of Africa, including data from Malawi. Squares represent each individual study prevalence with its area being proportional to the weight the study received in the meta‐analysis and horizontal lines showing the 95% CI's. Diamonds represent the combined estimates for each region with its width representing the 95% CI.

At stage 1, 766 PubMed publications on HTLV in African countries were identified. At stage 2, all publications that met the outlined inclusion/exclusion criteria were collected. Specific effort was made to extract data on women and children only from the manuscripts. Data were available for 24 countries for women and eight for children and were published between 1988 and 2014 for women and between 1988 and 1999 for children. Thirty‐four studies had data for cohorts of healthy volunteers, three for blood donors, twenty‐two for antenatal care (ANC) attendees and fifteen for sex workers. Mean sample size for women was 609 (range 26–2070) and 562 for children (range 46–1323). Some manuscripts gave data on multiple subgroups of study population therefore additional data sets were created. In the first instance, analysis was conducted on 74 data sets for women (Table 3, Figure 2) and 13 for children (Table 4, Figure 3) from a total of 68 publications, three of which contained data for children only (Figure 1, Table 2). Malawi findings were included in final analyses (Tables 3/4, Figures 2/3).

**Table 2:** Systematic review of all included manuscripts reporting African HTLV prevalence in healthy women and children

Reference	Year	Nation	Region	Cohort	Size	Prevalence %			
						HTLV‐1	HTLV‐2	Dual	Total
Larouze *et al*. 42	1985	Algeria	North	SW	140	0.00	0.00	0.00	0.00
Constantine *et al*. 43	1991	Egypt	North	SW	158	0.00	0.00	0.00	0.00
Larouze *et al*. 42	1985	Tunisia	North	HV	442	0.00	0.00	0.00	0.00
Fox *et al*. 44	1988	Djibouti	East	SW	327	1.22	0.00	0.00	1.22
Constantine *et al*. 45	1992	Djibouti	East	ANC	33	6.06	0.00	0.00	6.06
Andersson *et al* 46	1999	Eritrea	East	SW	97	0.00	2.06	0.00	2.06
				ANC	113	0.00	0.00	0.00	0.00
Ramos *et al*. 47	2011	Ethiopia	East	ANC	165	0.00	0.00	0.00	0.00
Ramos *et al*. 48	2012	Ethiopia	East	ANC	328	0.00	0.00	0.00	0.00
Scott *et al*. 49	1991	Somalia	East	SW	57	0.00	0.00	0.00	0.00
Bayley *et al*. 23	1985	Zambia	East	HV	132	0.00	0.00	0.00	0.00
Collenberg *et al*. 50	2006	Burkina Faso	West	ANC	492	1.02	0.00	0.00	1.02
Dumas *et al*. 51	1991	Benin	West	HV	1329	1.96	0.00	0.00	1.96
Houinato *et al*. 52	1996	Benin	West	HV	853	1.29	0.00	0.00	1.29
Ndumbe *et al*. 53	1992	Cameroon	West	ANC	170	0.59	0.00	0.00	0.59
Mauclere *et al*. 54	1993	Cameroon	West	SW	391	0.26	0.77	0.00	1.02
Mauclere *et al*. 55	1995	Cameroon	West	SW	332	0.90	0.30	0.00	1.20
Mauclere *et al*. 56	1997	Cameroon	West	HV	1977	1.52	0.00	0.00	1.52
Filippone *et al*. 33	2012	Cameroon	West	HV*	978	2.04	0.00	0.00	2.04
Gessain *et al*. 28	1993	CAR	West	HV	689	0.29	0.00	0.00	0.29
Wiktor *et al*. 57	1990	DR Congo	West	SW	377	3.18	0.00	0.00	3.18
Goubau *et al*. 58	1993	DR Congo	West	HV	1956	2.15	0.00	0.00	2.15
Jeannel *et al*. 59	1993	DR Congo	West	HV	352	0.85	0.00	0.00	0.85
Delaporte *et al*. 60	1995	DR Congo	West	ANC	1160	3.71	0.00	0.00	3.71
				SW	1183	7.27	0.00	0.00	7.27
Tuppin *et al*. 61	1996	DR Congo	West	ANC	2070	0.68	0.00	0.00	0.68
Schrijvers *et al*. 62	1991	Gabon	West	HV	651	6.76	0.00	0.00	6.76
Delaporte *et al*. 63	1993	Gabon	West	HV	30	16.67	0.00	0.00	16.67
Delaporte *et al*. 40	1993	Gabon	West	ANC	434	7.14	0.00	0.00	7.14
Le Hesran *et al*. 64	1994	Gabon	West	HV	505	13.07	0.00	0.00	13.07
Bertherat *et al*. 65	1998	Gabon	West	HV	275	7.27	1.09	0.00	8.36
Moynet *et al*. 66	2001	Gabon	West	HV	311	7.90	0.00	0.00	7.90
Etenna *et al*. 67	2008	Gabon	West	ANC	907	2.09	0.11	0.00	2.21
Biggar *et al*. 68	1993	Ghana	West	HV	1242	1.29	0.00	0.00	1.29
Apea‐Kubi *et al*. 69	2006	Ghana	West	ANC	294	2.72	0.00	0.00	2.72
Armah *et al*. 70	2006	Ghana	West	ANC	960	2.08	0.10	0.00	2.19
Jeannel *et al*. 71	1995	Guinea	West	HV	718	2.23	0.00	0.00	2.23
Naucler *et al*. 72	1992	Guinea‐Bissau	West	ANC	272	3.31	0.00	0.00	3.31
Norrgren *et al*. 73	1995	Guinea‐Bissau	West	HV	143	4.90	0.00	0.00	4.90
Andersson *et al*. 74	1997	Guinea‐Bissau	West	ANC	1231	2.19	0.08	0.00	2.27
Melbye *et al*. 75	1998	Guinea‐Bissau	West	HV	193	12.44	0.00	0.00	12.44
Larsen *et al*. 76	2000	Guinea‐Bissau	West	HV	1183	4.73	0.08	0.00	4.82
Holmgren *et al*. 77	2002	Guinea‐Bissau	West	HV	1605	5.79	0.00	0.00	5.79
Holmgren *et al*. 78	2003	Guinea‐Bissau	West	HV	816	10.17	0.00	0.00	10.17
Ariyoshi *et al*. 79	2003	Guinea‐Bissau	West	HV	105	10.48	0.00	0.00	10.48
Norrgren *et al*. 80	2008	Guinea‐Bissau	West	HV	1050	3.24	0.00	0.00	3.24
da Silva *et al*. 81	2009	Guinea‐Bissau	West	HV	1507	2.92	0.00	0.00	2.90
Ouattara *et al*. 82	1989	Ivory Coast	West	HV	594	2.02	0.00	0.00	2.02
				SW	149	2.68	0.00	0.00	2.68
Verdier *et al*. 83	1989	Ivory Coast	West	ANC	513	1.95	0.00	0.00	1.95
				SW	390	7.44	0.00	0.00	7.44
Dada *et al*. 84	1993	Nigeria	West	SW	885	2.82	0.00	0.00	2.82
Olaleye *et al*. 85	1994	Nigeria	West	SW	60	3.33	3.33	1.67	8.33
Olaleye *et al*. 86	1995	Nigeria	West	ANC	364	5.50	3.85	2.20	11.54
Olaleye *et al*. 39	1999	Nigeria	West	HV	460	4.35	1.09	0.00	5.43
Eltom *et al*. 87	2002	Nigeria	West	SW	863	3.24	0.00	0.00	3.24
Durojaiye *et al*. 88	2014	Nigeria	West	HV	26	0.00	0.00	0.00	0.00
Okoye *et al*. 89	2014	Nigeria	West	ANC	200	0.50	0.00	0.00	0.50
Larouze *et al*. 42	1985	Senegal	West	HV	237	0.00	0.00	0.00	0.00
Diop *et al*. 90	2006	Senegal	West	BD	1315	0.08	0.00	0.00	0.08
				ANC	178	1.78	0.00	0.00	1.78
Pepin *et al*. 91	1991	The Gambia	West	SW	354	10.45	0.00	0.00	10.45
Del Mistro *et al*. 37	1994	The Gambia	West	HV	909	1.21	0.00	0.00	1.21
Melo *et al*. 19	2000	Mozambique	South	ANC	132	0.76	0.00	0.00	0.76
Cunha *et al*. 20	2007	Mozambique	South	BD	576	1.22	0.00	0.00	1.22
Gudo *et al*. 21	2009	Mozambique	South	BD	373	0.80	0.00	0.00	0.80
Caterino‐de‐Araujo *et al*. 22	2010	Mozambique	South	HV	483	2.90	0.00	0.00	2.90
Steele *et al*. 92	1994	Namibia	South	HV*	30	3.33	0.00	0.00	3.33
Botha *et al*. 93	1985	South Africa	South	HV	211	5.21	0.00	0.00	5.21
				ANC	911	1.32	0.00	0.00	1.32
Bhigjee *et al*. 94	1993	South Africa	South	HV	527	1.90	0.00	0.00	1.90
Goubau *et al*. 58	1993	South Africa	South	ANC	428	0.23	0.00	0.00	0.23
Bhigjee *et al*. 95	1994	South Africa	South	HV	197	2.54	0.00	0.00	2.54
Taylor *et al*. 96	1996	South Africa	South	ANC	1259	0.56	0.00	0.00	0.56
Andersson *et al*. 46	1999	Eritrea	East	C	161	0.00	0.00	0.00	0.00
Steele *et al*. 92	1994	Namibia	South	C	244	0.82	0.00	0.00	0.82
Taylor *et al*. 96	1996	South Africa	South	C*	1323	0.38	0.00	0.00	0.38
Jeannel *et al*. 59	1993	DR Congo	West	C	715	1.40	0.00	0.00	1.40
Delaporte *et al*. 97	1988	Gabon	West	C	684	2.19	0.00	0.00	2.19
Delaporte *et al*. 40	1993	Gabon	West	C	610	2.79	0.00	0.00	2.79
Le Hesran *et al*. 64	1994	Gabon	West	C	378	3.70	0.00	0.00	3.70
Nyambi *et al*. 38	1996	Gabon	West	C	309	6.80	0.32	0.00	7.12
Verdier *et al*. 83	1989	Ivory Coast	West	C	364	1.37	0.00	0.00	0.00
Williams *et al*. 98	1993	Nigeria	West	C	46	6.52	0.00	0.00	6.52
Olaleye *et al*. 85	1994	Nigeria	West	C	1081	0.74	0.46	0.19	1.39
Olaleye *et al*. 39	1999	Nigeria	West	C	476	1.05	0.00	0.00	1.05
Del Mistro *et al*. 37	1994	The Gambia	West	C	916	0.11	0.00	0.00	0.11

DR Congo, Democratic Republic of Congo; HV, healthy volunteers; HV*, bushwomen; BD, blood donor; ANC, antenatal clinic; SW, sex worker; C, children, Children*, younger than 72 months of age.

**Table 3:** Prevalence of HTLV‐1 and HTLV‐2 in healthy adult women in four geographically defined African regions determined through meta‐analysis of eligible publications defined by the systematic review

Region (sample number)	Countries (sample number)	Without Malawi data		With Malawi data	
		% seroprevalence	95% CI	% seroprevalence	95% CI
North (3)	Algeria (1), Egypt (1), Tunisia (1)	0.0	0.0–1.1		
West and Central (52)	Benin (2), Burkina Faso (1), Cameroon (5), Central Africa Republic (1), DR Congo (6), Gabon (7), Gambia (2), Ghana (3), Guinea‐Bissau (10), Guinea (1), Ivory Coast (4), Nigeria (7), Senegal (3)	3.2	2.6–4.0		
East (8)	Djibouti (2), Eritrea (2), Ethiopia (2), Zambia (1) Somalia (1)	1.0	0.5–2.0		
East (9)	Djibouti (2), Eritrea (2), Ethiopia (2), Malawi (1), Zambia (1) Somalia (1)			1.2	0.6–2.3
Southern (11)	Mozambique (4), Namibia (1), South Africa (6)	1.6	1.0–2.5		

DR Congo, Democratic Republic of Congo.

**Table 4:** Prevalence of HTLV‐1 and HTLV‐2 in children in four geographically defined African regions determined through meta‐analysis of eligible publications defined by the systematic review

Region (sample number)	Countries (sample number)	Without Malawi data		With Malawi data	
		% seroprevalence	95% CI	% seroprevalence	95% CI
North (0)					
West and Central (10)	DR Congo (1), Gabon (4), Gambia (1), Ivory Coast (1), Nigeria (3)	2.0	1.2–3.4		
East (1) East (2)	Eritrea (1) Eritrea (1), Malawi (1)	0.3	0.0–4.7	1.3	0.2–7.1
Southern (2)	Namibia (1), South Africa (1)	0.5	0.2–1.0		

DR Congo, Democratic Republic of Congo.

We analysed data by country and region (North, West and Central, East and Southern Africa) but deliberately refrained from calculating a prevalence estimate for the African continent (Table 3, Figure 2). For women, the highest seroprevalence of HTLV was reported in West and Central Africa (3.2%, 95%CI 2.6‐4.0), which was also the source of the most studies (54/74 data sets, 13 countries). Limited data were available for children but West and Central Africa was again the most commonly studied region (10 of 13 data sets, 5 countries) with an estimated HTLV seroprevalence of 2.0% (95%CI 1.2‐3.4) (Table 4, Figure 3). Both for women and children, the most commonly reported subtype was HTLV‐1. HTLV‐1 and HTLV‐2 dual infections in healthy women and children were only reported in Nigeria, at 2.2% and 0.2%, respectively, and in sex workers at 1.67% (Table 2).

Assuming that sex workers may have an unusually high exposure risk we compared them to other women using logistic regression with robust standard errors allowing for the clustering in studies and adjusting for region. The odds ratio of HTLV for sex workers compared to all other women was 1.34 (95% CI 0.80–2.23, *P* = 0.3). Therefore, although they had a slightly higher risk of being positive for HTLV, this may have been due to chance variation; therefore, sex workers were not excluded from the analysis.

HTLV is more prevalent in sexually active and older women 2. Analysis of HTLV by age stratification could not be conducted as age was not available on all the mothers in our study; however, they were all sexually active and of reproductive age.

In light of the HTLV‐2 seroprevalence in our Malawi study, we were interested to know the seroprevalence of HTLV‐2 in Africa. HTLV‐2 had been observed in 11 of 74 data sets for women with stark regional variability: Eritrea 2.1%, Cameroon 0.3% and 0.8%, Gabon 1.1 and 0.1%, Ghana 0.1%, Guinea‐Bissau 0.1% (on two occasions), Nigeria 1.1% and 3.9% (Table 2). HTLV‐2 was concentrated in women of Pygmy origin. Two of 13 studies reported HTLV‐2 in children: Nigeria 0.5% and Gabon 0.3% (Table 2).

Focusing on countries neighbouring Malawi, HTLV‐2 was not observed in four studies originating from Mozambique (combined total sample size: 1564) 19, 20, 21, 22 nor in the only Zambian study 23; publications from Tanzania did not report confirmatory testing and were excluded from the meta‐analysis. Our observation of HTLV‐2 at 1.7% in women and 1.3% in children was high in comparison with the rest of Africa as well as neighbouring countries, but similar to Nigeria and Eritrea.

## Discussion

To our knowledge, this is the first report on the seroprevalence and type of HTLV in Malawi combining screening with robust confirmatory testing. We had access to stored sera of children with childhood cancers and their mothers as their controls who had taken part in a previous study 15. HTLV seroprevalence was 2.6% in mothers and 2.2% in children. HTLV‐2 was seen more commonly at 1.7% and 1.3% for mothers and children compared to 0.7% and 0.6% for HTLV‐1, respectively. None of the HTLV‐positive cases were hepatitis C 24 or HIV positive. High levels of HTLV intermediate screening results are commonly seen in African sera 25; only one of the 18 tested indeterminate samples could be resolved by DNA testing. Ten of 12 HTLV‐positive children had HTLV‐negative mothers.

In this retrospective study, the mothers were of child‐bearing age with known multiple pregnancies but we can only make assumptions about their HTLV acquisition. They could have acquired these viruses through: MTCT from their own mothers; unprotected sex; contact with non‐human primates (bites/hunting/butchering, pets), use of unsterile instrumentation (female genital mutilation [FGM], tattooing/piercing) or contaminated needles; unscreened blood products or other means not yet characterised. Bush‐meat hunting, heroin trafficking and FGM are prevalent in Malawi although not sufficiently surveyed 26. To our knowledge, no publications examining IVDU in Malawi are available, but a systematic review of data on IVDU in African populations showed that IVDU was more prevalent in adult males (66–94% of IVDU use) and almost exclusively seen in female sex workers 26. In our cohort, none of the HTLV‐positive mothers reported sex work or drug usage or a partner who had engaged in IVDU. The Malawian blood transfusion service (MBTS) tests blood donations for HIV, HBV, HCV, syphilis and malaria but not for HTLV (personal communication with Malawian blood transfusion services). Prior to the establishment of the MBTS, one study assessed the safety of blood donors in Malawi in 2001 14. Among 159 blood donations, the prevalence of HIV‐1 infection was 10.7%, 8.1% for HBV carriage, 6.8% for anti‐HCV and 2.5% for anti‐HTLV‐1.

The lack of concordance between mothers and their children remains unexplained; it might be an indication of horizontal transmission or the possibility that the child had been breastfed by someone other than their mother. The origin and route of transmission of the unusually high HTLV‐2 seroprevalence found in our study participants remains unknown and prospective study would allow verification and identification of infection source.

Our systematic review of HTLV in women and children confirmed a distribution of HTLV in large areas of Africa (West, Central, East and Southern Africa). HTLV was not detected in healthy women in the three eligible studies from North Africa (*n* = 740); this region seems to have a low HTLV seroprevalence, although this should be verified with additional larger prevalence studies.

The prevalence of HTLV‐1 in many areas of the world is poorly mapped and incidence of other subtypes is even less well known. Africa is thought to be endemic for HTLV 2, but prevalence data are only partially available or studies have only be conducted using ELISA without confirmation or typing. East Africa is especially under surveyed and HTLV untyped. Malawi is known for its high HIV‐1 prevalence (~11%) 27, but HTLV has only been reported in a single study with a reported seroprevalence of 2.5% in blood donors 14. The overall HTLV seroprevalence in our study was thus very similar to that found in this previous report. As typing was not performed in the study by Candotti *et al*., it is possible that HTLV‐2 was present in these blood donors.

In our systematic review only two other African countries reported high rates of HTLV‐2 seroprevalence: Nigeria and Eritrea, about 2000 miles apart. HTLV‐2 was not detected in Mozambique or Zambia, neighbouring countries of Malawi (Table 2). Worldwide, HTLV‐2 is endemic in the Americas and associated with IVDU 4, but in Africa it is endemic in West and Central Africa, as confirmed by our systematic review 28, 29, 30, 31, 32, 33, 34, 35, 36. None of the reviewed studies reported IVDU but mostly unprotected sex as the risk factor for HTLV acquisition.

Three manuscripts in our systematic review addressed HTLV MTCT 37, 38, 39, two of which also reported serodiscordance between mothers and their children. In the first of these studies, Del Mistro *et al*., identified an HTLV‐1 infected child, from a total of 916 children screened, whose mother had an indeterminate serological pattern and a negative HTLV PCR result 37. Secondly, Olaleye *et al*., reported five HTLV‐1‐positive children of 476 children from a total of 460 mothers in their study; none of the mothers of the infected children had HTLV 39. The other interesting feature of this study was that none of the 20 HTLV‐positive mothers had HTLV‐positive children. Our hypotheses as to how these childhood infections could have occurred align with those of the authors of these reports: infection of children with negative mothers may possibly be a result of repeat blood transfusions for a variety of conditions, including sickle cell disease and malaria as well as usage of unsterilised body scarification instruments 39, 40. We are aware that 18% of all the children in our study had historical hospital admission, in addition approximately 50% were hospital‐born and 11% had received blood transfusions. However, to our knowledge, none of the HTLV‐positive children in our study had received a blood transfusion. Breastfeeding is a potential virus transmission route if the children had been breastfed by an infected wet nurse other than their HTLV‐discordant or HTLV‐negative mother. We had no data on the sexual activity of the children but only one child was older than 13 years. Exposure to wildlife and primate bites are additional risk factors 41.

The strengths of this study were the large total and matched sample sizes, the combination of highly sensitive and specific screening and confirmation testing and the HTLV mapping of Malawi, an under surveyed country. The study has several limitations. We tested stored samples and, therefore, were unable to prospectively collect demographic and risk factor data specific to HTLV seroprevalence studies. None of the HTLV‐positive cases were HIV positive, which is interesting in itself considering that both share the same route of transmission. Samples were also a few years old which might have affected their reactivity. The cohort of children had cancer and antibody cross reactivity might have led to false positive results, although the highly specific confirmatory testing should negate this risk and HTLV infections were also found in the healthy women. Additional molecular testing of seropositive samples would have been ideal but we did not have access to the required DNA.

In our systematic review, we included data only on healthy women to best match our cohort of women and reduce co‐infection/co‐morbidity bias. There were very few published reports of childhood HTLV and we excluded those who were known HIV positive. We believe data from the 1980s are valuable, although an overestimation of HTLV prevalence is possible. Furthermore, because African HTLV seroprevalence studies are limited in their number, we did not exclude any samples based on study size but calculated regional estimates rather than national estimates in an attempt to reduce dependence upon studies with small cohorts. Inherent bias from small studies and those from the 1980s was reduced by including only publications that had used serological confirmatory testing. We used appropriate study weighting based on cohort size in our analyses and regional seroprevalence estimates.

In summary, our study highlights that HTLV is prevalent in Malawi. However, neither ATLL nor HAM/TSP has been reported so far. These HTLV‐1‐associated diseases are difficult to diagnose with high risk of mortality from ATLL before diagnosis has been made. They are, therefore, notoriously underreported unless specifically looked for. In addition, HTLV‐2 is not as pathogenic as HTLV‐1 and will be transmitted unknowingly from generation to generation.

Prospective field studies of HTLV as well as its associated diseases are needed in Malawi to allow health policy makers to develop practical and sustainable health policies with the long‐term aim of eradicating HLTV.
